# The Disinfection of Mail Matter

**Published:** 1884-09

**Authors:** 


### The Disinfection of Mail Matter.

While Asiatic cholera is prevailing to such an alarming extent in Europe, the advisability of disinfecting mail matter is being considered by our sanitary authorities. In some German States, the foreign mail is carried in tarred bags, numerous holes are pierced in letters and packages and the whole lot is fumigated. This means of disinfection has been ridiculed by Prof. Pettenkofer in his report on the subject to the Bavarian government. He claims that there is no evidence whatever to prove that contagious germs are imported by the mails, and characterizes all attempts in this direction as "Love's labor lost." In proof of his statements he shows that although the postal service has been greatly extended and accelerated during the last few years, the disease is not disseminated more rapidly than formerly. In spite of the increased communication with Calcutta and Bombay, places where cholera is never extinct, Europe is not visited by cholera more often than formerly. Besides this, men who handle the mails are not especially liable to infection. England, in 1872–4, was free from cholera, although the disease prevailed in many Continental countries, and the mails were not submitted to sanitary inspection. Pettenkofer's views seem to coincide with those of Florence Nightingale, who, although ignorant of the gyrations of germs, has had ample experience with cholera, and claims that disinfection is almost worthless, purity and cleanliness being the only means of resisting the invasions of this disease.

